# Multiple neonicotinoids in children’s cerebro-spinal fluid, plasma, and urine

**DOI:** 10.1186/s12940-021-00821-z

**Published:** 2022-01-11

**Authors:** Bernard Laubscher, Manuel Diezi, Raffaele Renella, Edward A. D. Mitchell, Alexandre Aebi, Matthieu Mulot, Gaëtan Glauser

**Affiliations:** 1 Departments of Paediatrics, Réseau Hospitalier Neuchâtelois, Neuchâtel, and Lausanne University Hospital and Lausanne University, Lausanne, Switzerland; 2 Department of Paediatrics, Lausanne University Hospital and Lausanne University, Lausanne, Switzerland; 3 Institute of Biology, University of Neuchâtel, Neuchâtel, Switzerland; 4 Institutes of Biology and Anthropology, University of Neuchâtel, Neuchâtel, Switzerland; 5 Neuchâtel Platform of Analytical Chemistry, University of Neuchâtel, Neuchâtel, Switzerland

**Keywords:** Neonicotinoid, *N*-desmethyl-acetamiprid, Pesticide, Child, Cerebro-spinal fluid

## Abstract

**Background:**

Neonicotinoids (NN) are selective neurotoxic pesticides that bind to insect but also mammal nicotinic acetycholine receptors (nAChRs). As the most widely used class of insecticides worldwide, they are ubiquitously found in the environment, wildlife, and foods, and thus of special concern for their impacts on the environment and human health. nAChRs are vital to proper brain organization during the prenatal period and play important roles in various motor, emotional, and cognitive functions. Little is known on children’s contamination by NN. In a pilot study we tested the hypothesis that children’s cerebro-spinal fluid (CSF) can be contaminated by NN.

**Methods:**

NN were analysed in leftover CSF, blood, and urine samples from children treated for leukaemias and lymphomas and undergoing therapeutic lumbar punctions. We monitored all neonicotinoids approved on the global market and some of their most common metabolites by ultra-high performance liquid chromatography-tandem mass spectrometry.

**Results:**

From August to December 2020, 14 children were consecutively included in the study. Median age was 8 years (range 3–18). All CSF and plasma samples were positive for at least one NN. Nine (64%) CSF samples and 13 (93%) plasma samples contained more than one NN. Thirteen (93%) CSF samples had *N*-desmethyl-acetamiprid (median concentration 0.0123, range 0.0024–0.1068 ng/mL), the major metabolite of acetamiprid. All but one urine samples were positive for ≥ one NN. A statistically significant linear relationship was found between plasma/urine and CSF *N*-desmethyl-acetamiprid concentrations.

**Conclusions:**

We have developed a reliable analytical method that revealed multiple NN and/or their metabolites in children’s CSF, plasma, and urine. Our data suggest that contamination by multiple NN is not only an environmental hazard for non-target insects such as bees but also potentially for children.

**Supplementary Information:**

The online version contains supplementary material available at 10.1186/s12940-021-00821-z.

## Background

In 2017, United Nations rapporteurs called for a new global treaty to regulate and phase out the use of hazardous pesticides in farming as chronic exposure to pesticides had been linked to various neurological disorders. Pregnant women, foetuses, and children were also considered as particularly vulnerable [1].

Residential, household or parental exposures to pesticides have been associated with young adult brain tumors, respectively childhood leukemia and paediatric non-central nervous system solid tumors [2–5].

Among pesticides, neonicotinoids (NN), which are selectively neurotoxic and bind to nicotinic acetycholine receptors (nAChRs), are of special concern for their impacts on the environment and human health since they are the most widely used class of insecticides worldwide [6] and are ubiquitously found in the environment [7], wildlife [8], and various foods [9, 10]. NN use has been restricted in some part of the world due to their significant toxicity to non-target insects such as bees [11].

In mammals, nAChRs are vital to proper brain organization during the prenatal period [12]. In addition, they play important roles in various motor, emotional, and cognitive functions [12–14]. Little is known on the effect of chronic human low-level exposure to nAChRs’ disrupters such as NN, which, especially in human foetuses’ and children’s developing brains, could potentially lead to later cerebral dysfunctions. In humans, NN have been associated with small-for-gestational-age neonates, congenital malformations, autism spectrum disorder, memory loss and finger tremor [15–19]. NN toxicological studies in rodents or mammals/human cell-lines have been shown to be cytotoxic, genotoxic, hepatotoxic, haematotoxic, nephrotoxic and potentially immunotoxic [20–23]. Among pesticides, NN definitely represent a potential significant public-health risk.

Data on NN distribution and metabolism in humans are scarce [24]. There are very few studies in children: six publications on NN urinary levels [19, 25–29] and one study on NN hair concentrations (6–83 years old people, of whom 28 were < 16 years) [30]. In China, NN residues in urine were found in 81% of 289 seven to 11 year old school children [29] and in 100% of 324 tested donors of all ages (1–97 years, 111 children < 18 years) [25]. In Japan, urinary *N*-desmethyl-acetamiprid (Desm-ACT) was detected in 14/57 (24·6%) very low birth weight infants within 48 h of birth [19]. This level of prevalence is worrying and calls for studies on other body compartments and on potential health impacts. There are no data on direct human brain exposure to NN and there is only one report on NN detection in human cerebrospinal fluid (CSF), in a post mortem of a male adult after voluntary and fatal imidacloprid ingestion [31]. Assessing NN presence in children’s brain tissue is ethically complicated and, currently, the best available surrogate for brain environment is CSF [32].

The general aims of this pilot study were to develop an analytical protocol to measure NN in children’s CSF and to assess if NN are present in children’s CSF. Our hypothesis was that NN could be found in children’s CSF, thereby representing a potential central nervous system (CNS) exposure.

## Methods

In a prospective paediatric observational study in Switzerland, we conducted a comparative analysis of CSF, blood and urine to develop and test the analytical method and compare the measured concentrations in these three body fluids.

### Patients and procedures

To study children’s CSF in an ethically acceptable way, we included, as a convenience sample, children with oncologic disease whose CSF had to be removed for clinical reasons in large enough volumes to allow aliquots to be collected for research purposes. All children were diagnosed and treated at the Department of paediatrics, Lausanne University Hospital, where they were consecutively included between August and December 2020. After initial diagnostic procedures, all children (0–18 years) presenting acute lymphoblastic (ALL), myeloblastic (AML) leukaemia or non-Hodgkin lymphoma (NHL) and were planned to receive routine intrathecal chemotherapy were approached and included in the study after informed consent was obtained. Intrathecal treatments consisted in removing up to 8 mL of CSF by lumbar tap under *conscious* sedation or anaesthesia before injecting 8–15 mL of various chemotherapeutic agents; two mL of CSF was reserved for routine chemical, biological, and cytological investigations and 2 mL of the leftover CSF was collected for NN analysis. On the same occasion, blood was drawn for clinical purposes from a subcutaneous implantable device. From the four mL drawn to rinse the device (internal volume ≤ 1.7 mL), the first 2 mL were discarded, and the following 2 mL were kept (3.2% Citrate S-Monovette®, Sarstedt AG, Sevelen Switzerland) for NN analysis. The patient also voided freely a 5–10 mL aliquot of urine in a clean tube. CSF and urine were initially kept in 15 mL polypropylene tubes. All samples were centrifuged, to collect plasma from the blood samples and to remove sediments from the CSF and urine samples. The supernatants were stored at − 80 °C.

### NN analysis

Aliquots of 0.2 mL of CSF, plasma or urine were placed into 2.0 mL microcentrifuge tubes, to which 580 μL of acetonitrile and 20 μL of a solution of isotopically labelled internal standards (IS) were added. The IS solution contained acetamiprid-d3 (CDN Isotopes, Pointe-Claire, Canada), clothianidin-d3 (CDN Isotopes), thiamethoxam-d4 (CDN Isotopes), thiacloprid-d4 (CDN Isotopes), imidacloprid-d4 (CDN Isotopes), dinotefuran-d3 (EQ Laboratories, Augsburg, Germany), and nitempyram-13C-d3 (Alsachim, Illkirch-Graffenstaden, France) at a concentration of 50 ng/mL in acetonitrile. The mixture was swiftly vortexed and ultrasonicated to precipitate proteins. After centrifugation, the supernatant was partially evaporated to a volume of ca. 50–100 μL, diluted with 950 μL of a 2% formic acid solution in water and submitted to a purification procedure using solid-phase extraction (SPE) cartridges. The SPE cartridge was first conditioned with 1 mL of methanol, equilibrated with 1 mL of 2% formic acid, loaded with the sample, washed with 1 mL of 0.1% formic acid and finally eluted with 1 mL of methanol. The fraction was evaporated to dryness and reconstituted in 200 μL of methanol 25% in water, ultrasonicated, centrifuged, and filtered through 13 mm hydrophilic PTFE filters. A 2.5 μL of the resulting extract was injected into an ultra-high performance liquid chromatography-tandem mass spectrometry (UHPLC-MS/MS) system composed of an Acquity UPLC I-Class and a TQ-XS triple quadrupole (Waters, Milford, MA). NN were detected and quantified according to Kammoun and colleagues^34^. All neonicotinoids and related insecticides approved on the global market and their most commonly detected metabolites were targeted: acetamiprid, clothianidin, thiamethoxam, thiacloprid, imidacloprid, dinotefuran, nitempyram, sulfoxaflor, flupyradifurone, Desm-ACT, imidacloprid-olefin (IMI-olefin), desnitro-imidacloprid (IMI-NH), and 6-chloronicotinic acid (6-CAN). The column used for the separation was an Acquity UPLC HSS T3 (2.1x100mm, Waters). Mobiles phases were milli-Q water + 0.05% formic acid + 1 mM ammonium formate (phase A) and acetonitrile + 0.05% formic acid (phase B). The gradient program started at 0% phase B and increased linearly to 37.5% in 7.5 min, followed by a rapid increase to 100% B in 0.5 min, wash at 100% B for 2 min and reequilibration at 0% B for 4 min. The MS source conditions were identical to those presented in Kammoun and colleagues [33] except that the MS was operated in both electrospray positive and negative ionization using a 20 ms polarity switching time. Neonicotinoids were monitored based on the multiple reaction monitoring (MRM) mode. Compound-dependent parameters are presented in supplementary Table 1.

### Method validation

The performances of the method in terms of precision (expressed as % relative standard deviation (RSD)) and accuracy (expressed in %) were evaluated by spiking a known amount of NN in the different matrices before sample preparation (final concentration of 1 ng/mL, *n* = 4 for each matrix). We could not perform validation at multiple concentrations due to too restricted sample availability. Coefficients of variations (%RSD) were below 10% for all analytes and accuracies always ranged between 80 and 110% except for desnitro-imidacloprid in CSF and imidacloprid-olefin in plasma for which lower but still acceptable accuracy values of 74.1 and 67.1%, respectively, were obtained (supplementary Table 2). The response function was established using 6-point calibration curves ranging between 0.005 and 10 ng/mL. Linear or quadratic curves weighted by 1/x were applied. Limits of quantification (LOQs) were evaluated as the concentrations giving signal-to-noise ratios of 10 in spiked samples. The method LOQs in CSF ranged between 1 and 20 pg/mL for all substances except 6-CAN for which the LOQ was 200 pg/mL. Plasma LOQs were similar to CSF’s but urine LOQs were generally higher due to increased background noise and matrix effects (supplementary Table 2). The specificity was determined by measuring blank solutions submitted to the entire preparation as well as spiked samples.

### Total CSF protein content

The total CSF protein concentration was measured using a pyrogallol red-based colorimetric assay (Randox Laboratories, Crumlin, UK) on a Cobas 8000® modular analyzer (Roche Diagnostics, Rotkreuz, Switzerland).

### Specific gravity

Urinary concentrations can vary widely over the period of elimination of xenobiotics and largely depend on fluid intake. The two most common methods for correction of metabolite concentrations in urine are the measure of specific gravity and that of creatinine [34]. The specific gravity of urine samples was measured using a digital Atago PEN urine specific gravity refractometer (Tokyo, Japan). In brief, an aliquot of urine (0.2 mL) was placed on a microscope slide and the pen tip put horizontally on the slide for about 2 seconds until the measurement was done.

### Statistical analyses

Statistical analyses were carried out in R statistical software [35]. To assess the relationship between NN concentration is CSF and plasma or urine, we first selected NNs for which the number of samples with a concentration above LOQ was greater than 10. We then computed the association between NN concentration in CSF vs plasma and CSF vs urine using standard OLS linear regressions. Regressions were built using the lm function from the stats package [36] through ggpubr [37]. We set alpha = 0.05 as threshold for any analysis.

As this pilot study was only meant to test the feasibility of the NN measurements in children’s CSF, the only patient’s characteristics that were obtained were genders and ages and they were not considered as relevant confounding factors from a medical standpoint.

The study was approved by our local Institutional Review Board (Commission cantonale d’éthique de la recherche sur l’être humain, CER-VD). All teenagers/parents gave their written consent.

## Results

Over a 4 months period, 14 children with ALL (*n* = 9) or AML (*n* = 2) or NHL (*n* = 3) were consecutively included. M/F ratio was 1.0, median age 8 years (range 3–18). All CSF total protein contents were within institutional normal ranges (150–450 mg/l).

**Fig. 1:**
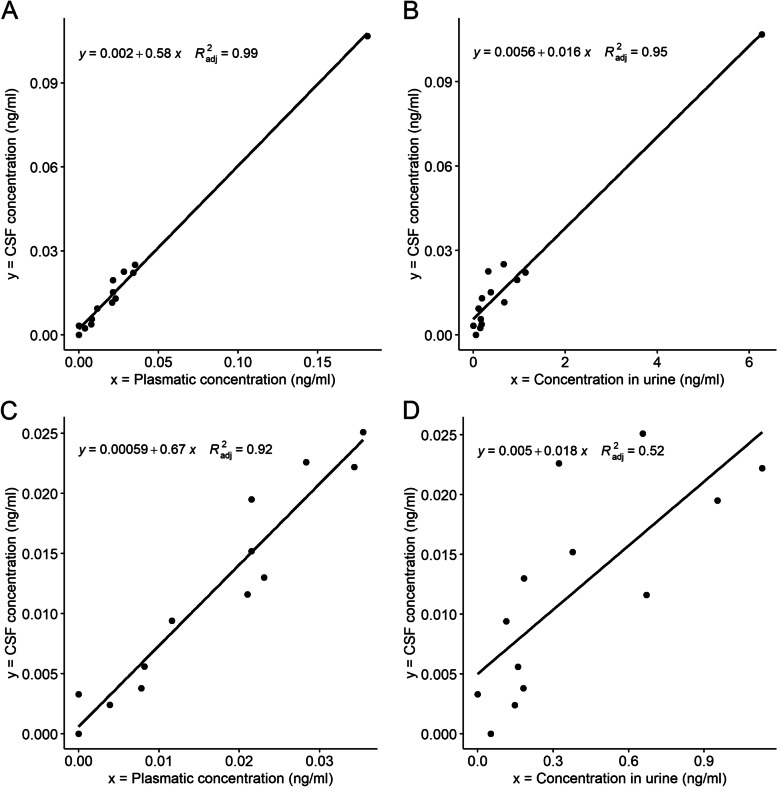
Cerebro-spinal fluid versus plasma (**A** and **C**) or urine (**B** and **D**) *N*-desmethyl-acetamiprid concentrations (ng/ml) of 14 children with haematological cancers. CSF, cerebro-spinal fluid. **A** and **B** *n* = 14. **C** and **D** *n* = 13, point with Cook distance > 1 removed. Urine concentrations corrected for specific gravity

All CSF presented detectable levels of at least one NN (Desm-ACT in 14/14, sulfoxaflor in 7/14, thiamethoxam in 6/14, and imidacloprid 2/14) (see Table 1 or supplementary Table 3 for detailed results). Five CSF contained a single NN (all with Desm-ACT), three contained two (two with Desm-ACT and sulfoxaflor and one with Desm-ACT and thiamethoxam), and six contained three (four with Desm-ACT, sulfoxaflor and thiamethoxam, one with Desm-ACT, sulfoxaflor and thiamethoxam and one with Desm-ACT, sulfoxaflor and imidacloprid). Thirteen out of 14 CSF had quantifiable levels of Desm-ACT (median concentration 0.0123, range 0.0024–0.1068 ng/mL). Four out of 14 CSF had quantifiable levels of sulfoxaflor (median concentration 0.0053, range 0.0024–0.0124 ng/mL). Three out of 14 CSF had quantifiable levels of thiamethoxam (median concentration 0.0196, range 0.0054–0.0765 ng/mL). One out of 14 CSF had quantifiable level of imidacloprid (0.0153 ng/mL). All plasma samples were positive for at least one NN: one with only thiamethoxam, six with two (three with sulfoxaflor and Desm-ACT, two with imidacloprid and Desm-ACT, one with thiamethoxam and Desm-ACT), five with three (two with thiametoxam, sulfoxaflor and Desm-ACT, two with thiametoxam, imidacloprid and Desm-ACT, one with imidacloprid, sulfoxaflor and Desm-ACT) and two with four (both with thiamethoxam, imidacloprid, sulfoxaflor and Desm-ACT). Thirteen out of 14 plasma samples had Desm-ACT (12 of which with quantifiable levels, median concentration 0.0213, range 0.0039–0.1812 ng/mL). All but one urine samples were positive for at least one NN (seven with one, four with two, one with three and one with four). Thirteen out of 14 urines had Desm-ACT (median concentration 0.1925, range 0.0378–6.774 ng/mL). Significant linear relationships were found between CSF and plasma/urine Desm-ACT concentrations (see Fig. 1). Other NN did not meet the inclusion criteria of a minimum of 10 samples with a concentration above LOQ. One data point had a Cook distance greater than one in both models (CSF vs plasma and CSF vs urine), with a Desm-ACT concentration in urine greater than 6 ng/mL. We computed new models excluding this point that were very similar to the full ones, with the exception that the intercept became not significant. All models were significant. Full details on the models are presented in supplementary material (supplementary Figs. 1 and 2, supplementary Tables 4 and 5).

**Table 1:** Summary of neonicotinoids and neonicotinoid metabolites in cerebro-spinal fluid, plasma and urine of 14 children with haematological cancers

	Cerebro-spinal fluid						Plasma						Urine^a^					
	Number samples			Min conc. (ng/mL)	Max conc. (ng/mL)	Median conc. (ng/mL, of >LOQ)	Number samples			Min conc. (ng/mL)	Max conc. (ng/mL)	Median conc. (ng/mL, of >LOQ)	Number samples			Min conc. (ng/mL)	Max conc. (ng/mL)	Median conc. (ng/mL, of >LOQ)
	< LOD	LOD < x < LOQ	> LOQ				< LOD	LOD < x < LOQ	> LOQ				< LOD	LOD < x < LOQ	> LOQ			
Thiamethoxam	8 (57%)	3 (21%)	3 (21%)	< LOD	0.0765	0.0196	6 (43%)	1 (7%)	7 (50%)	< LOD	0.4072	0.0203	14 (100%)	0	0	< LOD	< LOD	< LOD
Clothianidin	14 (100%)	0	0	< LOD	< LOD	< LOD	14 (100%)	0	0	< LOD	< LOD	< LOD	14 (100%)	0	0	< LOD	< LOD	< LOD
Imidacloprid	12 (86%)	1 (7%)	1 (7%)	< LOD	0.0153	0.0153	7 (50%)	6 (43%)	1 (7%)	< LOD	0.0216	0.0216	12 (86%)	1 (7%)	1 (/%)	< LOD	0.1208	0.1208
Acetamiprid	14 (100%)	0	0	< LOD	< LOD	< LOD	14 (100%)	0	0	< LOD	< LOD	< LOD	14 (100%)	0	0	< LOD	< LOD	< LOD
Thiacloprid	14 (100%)	0	0	< LOD	< LOD	< LOD	14 (100%)	0	0	< LOD	< LOD	< LOD	14 (100%)	0	0	< LOD	< LOD	< LOD
Flupyradifurone	14 (100%)	0	0	< LOD	< LOD	< LOD	14 (100%)	0	0	< LOD	< LOD	< LOD	14 (100%)	0	0	< LOD	< LOD	< LOD
Sulfoxaflor	7 (50%)	3 (21%)	4 (29%)	< LOD	0.0124	0.0053	6 (43%)	3 (21%)	5 (36%)	< LOD	0.0031	0.0015	10 (71%)	4 (29%)	0	< LOD	<LOQ	< LOD
Dinotefuran)	14 (100%)	0	0	< LOD	< LOD	< LOD	14 (100%)	0	0	< LOD	< LOD	< LOD	14 (100%)	0	0	< LOD	< LOD	< LOD
Nitempyram	14 (100%)	0	0	< LOD	< LOD	< LOD	14 (100%)	0	0	< LOD	< LOD	< LOD	14 (100%)	0	0	< LOD	< LOD	< LOD
*N*-desmethyl-acetamiprid	0	1 (7%)	13 (93%)	<LOQ	0.1068	0.0130	1 (7%)	1 (/%)	12 (86%)	< LOD	0.1812	0.0215	1 (7%)	0	13 (93%)	< LOD	6.2753	0.3235
Desnitro-Imidacloprid	14 (100%)	0	0	< LOD	< LOD	< LOD	14 (100%)	0	0	< LOD	< LOD	< LOD	14 (100%)	0	0	< LOD	< LOD	< LOD
Imidacloprid-olefin	14 (100%)	0	0	< LOD	< LOD	< LOD	14 (100%)	0	0	< LOD	< LOD	< LOD	11 (79%)	1 (7%)	2 (14%)	< LOD	0.2357	0.2331
6-Chloronicotinic acid	14 (100%)	0	0	< LOD	< LOD	< LOD	14 (100%)	0	0	< LOD	< LOD	< LOD	14 (100%)	0	0	< LOD	< LOD	< LOD

*LOD* limit of detection, *LOQ* limit of quantification; ^a^Corrected for specific gravity

## Discussion

Our study offers two new perspectives on paediatric public-health research: First, it shows that NN detection in children CSF is technically feasible. Second, it reveals that multiple NN can contaminate children’s CSF and that, in the case of Desm ACT, plasma and - to a lesser extent - urine concentrations correlate with CSF concentrations.

To our knowledge, no study has reported a method for the analysis of low concentrations of NNs in human CSF and their comparison with those in plasma and urine from the same individuals. Thus, we first had to develop a reliable methodology to measure neonicotinoids and their metabolites in these human fluids. The use of very small left-over volumes (0.2 mL) alleviates the difficulties related to CSF collection. However, starting from small sample volumes may have an impact on positive detection rates, which are often correlated to the method sensitivity [10]. Therefore, a sensitive analytical protocol able to detect traces of NN had to be developed. Using a combination of a two-step efficient clean-up (protein precipitation followed by solid-phase extraction) and state-of-the-art analysis, we were able to set quantification limits in CSF and plasma ranging between 1 and 20 pg/mL for all NN and their metabolites but one (6-CAN). In urine, LOQs generally ranged between 5 and 50 pg/mL, which is comparable or better than those from recent studies that started from higher or similar volumes of urine [19, 27, 38, 39]. In accordance with previous paediatric studies, our work also shows the importance to monitor as many molecules as possible, including the metabolites of parent pesticides [19, 27, 28, 38]. Had we chosen not to measure Desm-ACT, 7/14 children’s CSF would have been free of any NN, thus falsely minimizing their degree of contamination by NN. In children, only four studies reported on urinary Desm-ACT median concentrations which ranged between 0.048 ng/mL and 1.35 ng/mL [19, 27, 28, 38]. Our values (median concentration of 0.19 ng/mL) thus corroborate those found in earlier studies. We are not aware of any other studies on NN levels in paediatric body fluids. In 2021, Xu et al. reported on paired urine and blood NN profiles in 196 healthy young adults from China. They found urinary and blood Desm-ACT median concentrations of 0.35 and 0.58 ng/mL respectively, which are similar for urine but much higher for plasma than those found in our subjects (0.32 and 0.02 ng/mL, respectively) [40].

Our findings suggest that ALL, AML or NHL children living in Switzerland are exposed to multiple NN. Our study population was small and highly selected and is thus not representative of a large paediatric population, with or without a haematological cancer. As no control population of healthy children was included, we could not analyse if these multiple exposures to NN were specific to children with leukaemia/lymphoma. As we did not study potential exposures to any dietary pesticides by food or residential proximity to NN treated crops/livestock/pets, we could not analyse if these multiple exposures to NN were associated with increased individual environmental risks. The exposure to multiple NN in all included patients could represent the tip of the iceberg of a larger, but not analysed, exposure to pesticides. Although the Environmental Protection Agency has classified acetamiprid, clothianidin, dinotefuran, imidacloprid, and thiamethoxam as “Not Likely to be Carcinogenic to Humans” [41], and whilst one could argue that chronic exposure in children is too short to raise risks of secondary, non-genetic, cancers, exposures to pesticides - as a broad toxicological group - have been associated with children haematological cancer^3,4^ or CNS/non CNS solid tumors^2,5^. Such exposome outcomes to pesticides hazards have also recently been reported for neurodevelopmental issues [42].

To our knowledge, this is the first study on the presence of NN in humans’ CSF. Surprisingly, we observed remarkable differences in concentrations between acetamiprid and its metabolite Desm-ACT in CSF. We have found no data on absorption and distribution of NN and their metabolites in humans. Acetamiprid and Desm-ACT are structurally very similar and have comparable hydrophobicity (calculated logP 1.4 and 1.2, respectively), thus they should theoretically be found in the same body compartments. While we cannot totally exclude a Desm-ACT specific blood-to-CSF transfer, the observed differences in concentrations may be better explained by a prolonged half-life of Desm-ACT compared to ACT in the context of low-dose chronic exposure, resulting in the detection of the former only. The normal CSF total protein concentrations in the 14 studied patients argues against a meningeal inflammation and, thus, against an increased blood-to-CSF permeability such as in bacterial meningitis [43].

## Conclusions

Our work describes a reliable analytical procedure to measure NN in human CSF. Our data show that children with ALL, AML and NHL living in Switzerland are exposed to multiple NN. They also show that Desm-ACT freely diffuses into children’s CSF and that other NN than Desm-ACT are to be found in childrens’ CSF. No data exist on human blood-to-brain NN transfer, via the BBB or CSF-to-brain trans-neuroependymal route. The latter’s ultrastructure and ontogeny [44] as well as the now established presence of NN in children’s CSF warrants caution about a potential direct effect of NN on the CNS. The vulnerability of the maturing brain to NN could lie in a potential chronic low-dose exposure during a very susceptible developmental period. Exposome medium-term studies, from foetal life to late childhood, similar to the Helix cohort [45], are needed to better delineate potential links between NN exposures and childhood cancers. Organophosphate pesticides were measured in two sub-groups of the Helix cohort: it could be meaningful to add NN determination in the same samples. A larger study to collect more CSF/plasma/urine samples in children could yield more data on various NN levels in CSF/plasma/urine to allow a sounder analysis of blood-to-CSF or urine-to-CSF NN levels ratio.

## Supplementary Information

**Additional file 1: Supplementary Table 1.** UHPLC-MS/MS compound-specific parameters used for the quantification of neonicotinoids. RT, retention time; ESI, electrospray; CV, cone voltage; CE, collision energy.

**Additional file 2: Supplementary Table 2.** Validation parameters for the different analytes in the different matrices. IS; internal standard; LOQ limit of quantification; CSF cerebrospinal fluid.

**Additional file 3: Supplementary Table 3.** Detailed results of neonicotinoids and neonicotinoid metabolites in cerebro-spinal fluid, plasma and urine of 14 children with haematological cancers. CSF, cerebro-spinal fluid; LOD, limit of detection; LOQ, limit of quantification; * Corrected for specific gravity.

**Additional file 4: Supplementary Figure 1.** (CSF occurence). Number of samples with neonicotinoids > lower limit of quantification, per neonicotinoid, in cerebrospinal fluid in 14 children with haematological cancers. NN, neonicotinoid; CSF, cerebro-spinal fluid; LOQ, lower limit of quantification.

**Additional file 5: Supplementary Figure 2.** (plasma occurence). Number of samples with neonicotinoids > lower limit of quantification, per neonicotinoid, in plasma in 14 children with haematological cancers. NN, neonicotinoid; LOQ, lower limit of quantification.

**Additional file 6: Supplementary Table 4.** (models summary). Summary statistics of the regression models, with or without extreme value (cook distance > 1).

**Additional file 7: Supplementary Table 5.** (models coefficients). Coefficient and associated *p*-values of the regression models, with or without extreme value (Cook distance > 1).

## Data Availability

All data generated or analyzed during this study are included in this published article and its supplementary information files (Additional file, detailed results of NN analysis in CSF, plasma and urine).

## Abbreviations

CNS: Central nervous system
CSF: Cerebro-spinal fluid
Desm-ACT: *N*-desmethyl-acetamiprid
IMI-NH: Desnitro-imidacloprid
IMI-olefin: Imidacloprid-olefin
NN: Neonicotinoids
6-CAN: 6-chloronicotinic acid
