# Supplementary material for: Multiple neonicotinoids in children’s cerebro-spinal fluid, plasma, and urine
Source: Environ Health. 2022 Jan 11;21:10. doi: 10.1186/s12940-021-00821-z (PMC8750865; doi:10.1186/s12940-021-00821-z)
Supplement: Supplementary file 1 — Additional file 1: Supplementary Table 1. UHPLC-MS/MS compound-specific parameters used for the quantification of neonicotinoids. RT, retention time; ESI, electrospray; CV, cone voltage; CE, collision energy. [file 12940_2021_821_MOESM1_ESM.docx]

Table title: UHPLC-MS/MS compound-specific parameters used for the quantification of neonicotinoids

Table legend: RT, retention time; ESI, electrospray; CV, cone voltage; CE, collision energy

| Analyte | RT (min) | ESI polarity | CV  (V) | Transition 1  (quantifier) | CE 1  (V) | Transition 2  (qualifier) | CE 2  (V) |
| --- | --- | --- | --- | --- | --- | --- | --- |
| Dinotefuran | 3.63 | pos | 30 | 203 → 113 | 5 | 203 → 157 | 10 |
| Dinotefuran d-3 | 3.61 | pos | 30 | 206 → 160 | 5 | - | - |
| Nitenpyram | 4.25 | pos | 25 | 271 → 56 | 10 | 271 → 126 | 27 |
| Nitenpyram ^13^C-d3 | 4.24 | pos | 25 | 275 → 134 | 10 | - | - |
| Thiamethoxam | 5.09 | pos | 25 | 292 → 211 | 12 | 292 → 132 | 20 |
| Thiamethoxam d-3 | 5.07 | pos | 25 | 295 → 214 | 12 | - | - |
| Clothianidin | 5.73 | pos | 30 | 250 → 169 | 12 | 250 → 132 | 12 |
| Clothianidin d-3 | 5.71 | pos | 30 | 253 → 172 | 13 | - | - |
| Imidacloprid | 6.02 | pos | 35 | 256 → 209 | 15 | 256 → 175 | 20 |
| Imidacloprid d-4 | 6.00 | pos | 35 | 260 → 213 | 15 | - | - |
| Desnitro-imidacloprid | 3.41 | pos | 35 | 211 → 126 | 24 | 211→ 90 | 34 |
| Imidacloprid-olefin | 4.98 | pos | 10 | 254 → 205 | 15 | 254 → 171 | 18 |
| Acetamiprid | 6.44 | pos | 40 | 223 → 126 | 20 | 223 → 56 | 14 |
| Acetamiprid d-3 | 6.42 | pos | 40 | 226 → 126 | 20 | - | - |
| Desmethyl-acetamiprid | 5.85 | pos | 25 | 209 → 126 | 16 | 209 → 290 | 31 |
| Flupyradifurone | 7.02 | pos | 25 | 289 → 126 | 20 | 289 → 90 | 40 |
| Sulfoxaflor | 7.39 | pos | 40 | 174 → 154 | 16 | 174 → 104 | 25 |
| Thiacloprid | 7.40 | pos | 40 | 253 → 126 | 20 | 253 → 90 | 34 |
| Thiacloprid d-4 | 7.38 | pos | 40 | 257 → 126 | 20 | - | - |
| Chloronicotinic acid | 5.21 | pos/neg | 15/10 | 158 → 122 (+) | 18 | 156 → 112 (-) | 10 |
