# Supplementary material for: Multiple neonicotinoids in children’s cerebro-spinal fluid, plasma, and urine
Source: Environ Health. 2022 Jan 11;21:10. doi: 10.1186/s12940-021-00821-z (PMC8750865; doi:10.1186/s12940-021-00821-z)
Supplement: Supplementary file 2 — Additional file 2: Supplementary Table 2. Validation parameters for the different analytes in the different matrices. IS; internal standard; LOQ limit of quantification; CSF cerebrospinal fluid. [file 12940_2021_821_MOESM2_ESM.docx]

Table title: validation parameters for the different analytes in the different matrices

Table legend: IS; internal standard; LOQ limit of quantification; CSF cerebrospinal fluid

| Analyte | IS | Matrix | Precision (RSD%) | Accuracy (%) | LOQ (pg/mL) |
| --- | --- | --- | --- | --- | --- |
| Thiamethoxam | Thiamethoxam-d3 | CSF | 1.65 | 98.4 | 2 |
|  |  | Plasma | 1.89 | 104.2 | 2 |
|  |  | Urine | 1.69 | 105.6 | 20 |
| Clothianidin | Clothianidin-d3 | CSF | 2.09 | 91.5 | 4 |
|  |  | Plasma | 1.32 | 97.5 | 4 |
|  |  | Urine | 2.81 | 105.7 | 20 |
| Imidacloprid | Imidacloprid-d4 | CSF | 0.60 | 97.4 | 4 |
|  |  | Plasma | 1.81 | 102.6 | 4 |
|  |  | Urine | 3.23 | 108.9 | 20 |
| Desnitro-imidacloprid | Thiamethoxam-d3 | CSF | 3.36 | 74.1 | 15 |
|  |  | Plasma | 4.03 | 84.6 | 15 |
|  |  | Urine | 5.54 | 107.4 | 40 |
| Imidacloprid-olefin | Clothianidin-d3 | CSF | 4.41 | 89.2 | 10 |
|  |  | Plasma | 4.54 | 67.1 | 15 |
|  |  | Urine | 9.20 | 81.9 | 150 |
| Acetamiprid | Acetamiprid-d3 | CSF | 1.52 | 97.6 | 1 |
|  |  | Plasma | 1.19 | 102.4 | 2 |
|  |  | Urine | 1.33 | 107.1 | 10 |
| Desmethyl-acetamiprid | Acetamiprid-d3 | CSF | 1.62 | 97.5 | 4 |
|  |  | Plasma | 1.48 | 101.5 | 4 |
|  |  | Urine | 6.94 | 105.6 | 10 |
| Thiacloprid | Thiacloprid-d3 | CSF | 1.62 | 97.5 | 1 |
|  |  | Plasma | 1.86 | 102.2 | 2 |
|  |  | Urine | 0.84 | 104.1 | 5 |
| Flupyradifurone | Acetamiprid-d3 | CSF | 1.68 | 93.8 | 5 |
|  |  | Plasma | 0.87 | 94.8 | 5 |
|  |  | Urine | 7.60 | 96.1 | 25 |
| Sulfoxaflor | Thiacloprid-d3 | CSF | 2.75 | 103.1 | 1 |
|  |  | Plasma | 1.88 | 106.1 | 2 |
|  |  | Urine | 4.59 | 93.7 | 10 |
| Dinotefuran | Dinotefuran-d3 | CSF | 2.67 | 97.1 | 20 |
|  |  | Plasma | 1.94 | 96.8 | 25 |
|  |  | Urine | 2.36 | 104.5 | 50 |
| Nitempyram | Nitempyram-13C-d3 | CSF | 0.98 | 98.4 | 15 |
|  |  | Plasma | 0.33 | 101.2 | 25 |
|  |  | Urine | 2.82 | 108.3 | 40 |
| Chloronicotinic acid | Clothianidin-d3 | CSF | 5.01 | 88.3 | 200 |
|  |  | Plasma | 2.15 | 94.6 | 200 |
|  |  | Urine | 7.21 | 90.5 | 1000 |
