# Supplementary material for: Multiple neonicotinoids in children’s cerebro-spinal fluid, plasma, and urine
Source: Environ Health. 2022 Jan 11;21:10. doi: 10.1186/s12940-021-00821-z (PMC8750865; doi:10.1186/s12940-021-00821-z)
Supplement: Supplementary file 3 — Additional file 3: Supplementary Table 3. Detailed results of neonicotinoids and neonicotinoid metabolites in cerebro-spinal fluid, plasma and urine of 14 children with haematological cancers. CSF, cerebro-spinal fluid; LOD, limit of detection; LOQ, limit of quantification; * Corrected for specific gravity. [file 12940_2021_821_MOESM3_ESM.docx]

Table title: Detailed results of neonicotinoids and neonicotinoid metabolites in cerebro-spinal fluid, plasma and urine of 14 children

Table legend: CSF, cerebro-spinal fluid; LOD, limit of detection; LOQ, limit of quantification; * Corrected for specific gravity

| Patient nr. | Matrix | Thiamethoxam (ng/mL) | Clothianidin (ng/mL) | Imidacloprid (ng/mL) | Acetamiprid (ng/mL) | Thiacloprid (ng/mL) | Flupyradifurone (ng/mL) | Sulfoxaflor (ng/mL) | Dinotefuran (ng/mL) | Nitempyram (ng/mL) | Acetamiprid-desmethyl (ng/mL) | Desnitro-Imidacloprid (ng/mL) | Imidacloprid-olefin (ng/mL) | 6-Chloronicotinic acid (ng/mL) |
| --- | --- | --- | --- | --- | --- | --- | --- | --- | --- | --- | --- | --- | --- | --- |
| 1 | CSF | < LOD | < LOD | < LOD | < LOD | < LOD | < LOD | < LOD | < LOD | < LOD | 0.1068 | < LOD | < LOD | < LOD |
| 2 | CSF | < LOD | < LOD | < LOD | < LOD | < LOD | < LOD | 0.0124 | < LOD | < LOD | 0.0038 | < LOD | < LOD | < LOD |
| 3 | CSF | LOD<x< LOQ | < LOD | < LOD | < LOD | < LOD | < LOD | 0.0024 | < LOD | < LOD | 0.0094 | < LOD | < LOD | < LOD |
| 4 | CSF | LOD<x< LOQ | < LOD | < LOD | < LOD | < LOD | < LOD | LOD<x< LOQ | < LOD | < LOD | 0.0130 | < LOD | < LOD | < LOD |
| 5 | CSF | < LOD | < LOD | 0.0153 | < LOD | < LOD | < LOD | LOD<x< LOQ | < LOD | < LOD | 0.0056 | < LOD | < LOD | < LOD |
| 6 | CSF | 0.0196 | < LOD | < LOD | < LOD | < LOD | < LOD | 0.0071 | < LOD | < LOD | 0.0024 | < LOD | < LOD | < LOD |
| 7 | CSF | < LOD | < LOD | < LOD | < LOD | < LOD | < LOD | < LOD | < LOD | < LOD | 0.0195 | < LOD | < LOD | < LOD |
| 8 | CSF | 0.0054 | < LOD | < LOD | < LOD | < LOD | < LOD | < LOD | < LOD | < LOD | LOD<x< LOQ | < LOD | < LOD | < LOD |
| 9 | CSF | LOD<x< LOQ | < LOD | LOD<x< LOQ | < LOD | < LOD | < LOD | < LOD | < LOD | < LOD | 0.0222 | < LOD | < LOD | < LOD |
| 10 | CSF | 0.0765 | < LOD | < LOD | < LOD | < LOD | < LOD | LOD<x< LOQ | < LOD | < LOD | 0.0251 | < LOD | < LOD | < LOD |
| 11 | CSF | < LOD | < LOD | < LOD | < LOD | < LOD | < LOD | 0.0035 | < LOD | < LOD | 0.0226 | < LOD | < LOD | < LOD |
| 12 | CSF | < LOD | < LOD | < LOD | < LOD | < LOD | < LOD | < LOD | < LOD | < LOD | 0.0116 | < LOD | < LOD | < LOD |
| 13 | CSF | < LOD | < LOD | < LOD | < LOD | < LOD | < LOD | < LOD | < LOD | < LOD | 0.0152 | < LOD | < LOD | < LOD |
| 14 | CSF | < LOD | < LOD | < LOD | < LOD | < LOD | < LOD | < LOD | < LOD | < LOD | 0.0033 | < LOD | < LOD | < LOD |
|  |  |  |  |  |  |  |  |  |  |  |  |  |  |  |
| 1 | Plasma | < LOD | < LOD | LOD<x< LOQ | < LOD | < LOD | < LOD | LOD<x< LOQ | < LOD | < LOD | 0.1812 | < LOD | < LOD | < LOD |
| 2 | Plasma | < LOD | < LOD | < LOD | < LOD | < LOD | < LOD | 0.0015 | < LOD | < LOD | 0.0078 | < LOD | < LOD | < LOD |
| 3 | Plasma | LOD<x< LOQ | < LOD | LOD<x< LOQ | < LOD | < LOD | < LOD | 0.0012 | < LOD | < LOD | 0.0116 | < LOD | < LOD | < LOD |
| 4 | Plasma | 0.0203 | < LOD | < LOD | < LOD | < LOD | < LOD | 0.0013 | < LOD | < LOD | 0.0231 | < LOD | < LOD | < LOD |
| 5 | Plasma | < LOD | < LOD | 0.0216 | < LOD | < LOD | < LOD | < LOD | < LOD | < LOD | 0.0082 | < LOD | < LOD | < LOD |
| 6 | Plasma | < LOD | < LOD | LOD<x< LOQ | < LOD | < LOD | < LOD | < LOD | < LOD | < LOD | 0.0039 | < LOD | < LOD | < LOD |
| 7 | Plasma | 0.0040 | < LOD | < LOD | < LOD | < LOD | < LOD | < LOD | < LOD | < LOD | 0.0215 | < LOD | < LOD | < LOD |
| 8 | Plasma | 0.0101 | < LOD | LOD<x< LOQ | < LOD | < LOD | < LOD | < LOD | < LOD | < LOD | LOD<x< LOQ | < LOD | < LOD | < LOD |
| 9 | Plasma | 0.0030 | < LOD | LOD<x< LOQ | < LOD | < LOD | < LOD | < LOD | < LOD | < LOD | 0.0343 | < LOD | < LOD | < LOD |
| 10 | Plasma | < LOD | < LOD | < LOD | < LOD | < LOD | < LOD | 0.0021 | < LOD | < LOD | 0.0354 | < LOD | < LOD | < LOD |
| 11 | Plasma | 0.0628 | < LOD | < LOD | < LOD | < LOD | < LOD | 0.0031 | < LOD | < LOD | 0.0283 | < LOD | < LOD | < LOD |
| 12 | Plasma | < LOD | < LOD | < LOD | < LOD | < LOD | < LOD | LOD<x< LOQ | < LOD | < LOD | 0.0210 | < LOD | < LOD | < LOD |
| 13 | Plasma | 0.0786 | < LOD | LOD<x< LOQ | < LOD | < LOD | < LOD | LOD<x< LOQ | < LOD | < LOD | 0.0215 | < LOD | < LOD | < LOD |
| 14 | Plasma | 0.4072 | < LOD | < LOD | < LOD | < LOD | < LOD | < LOD | < LOD | < LOD | < LOD | < LOD | < LOD | < LOD |
|  |  |  |  |  |  |  |  |  |  |  |  |  |  |  |
| 1 | Urine* | < LOD | < LOD | < LOD | < LOD | < LOD | < LOD | < LOD | < LOD | < LOD | 6.2753 | < LOD | < LOD | < LOD |
| 2 | Urine* | < LOD | < LOD | < LOD | < LOD | < LOD | < LOD | LOD<x< LOQ | < LOD | < LOD | 0.1817 | < LOD | < LOD | < LOD |
| 3 | Urine* | < LOD | < LOD | < LOD | < LOD | < LOD | < LOD | < LOD | < LOD | < LOD | 0.1133 | < LOD | < LOD | < LOD |
| 4 | Urine* | < LOD | < LOD | < LOD | < LOD | < LOD | < LOD | < LOD | < LOD | < LOD | 0.1846 | < LOD | < LOD | < LOD |
| 5 | Urine* | < LOD | < LOD | 0.1208 | < LOD | < LOD | < LOD | LOD<x< LOQ | < LOD | < LOD | 0.1609 | < LOD | 0.2357 | < LOD |
| 6 | Urine* | < LOD | < LOD | < LOD | < LOD | < LOD | < LOD | < LOD | < LOD | < LOD | 0.1480 | < LOD | < LOD | < LOD |
| 7 | Urine* | < LOD | < LOD | < LOD | < LOD | < LOD | < LOD | < LOD | < LOD | < LOD | 0.9529 | < LOD | < LOD | < LOD |
| 8 | Urine* | < LOD | < LOD | < LOD | < LOD | < LOD | < LOD | < LOD | < LOD | < LOD | 0.0522 | < LOD | < LOD | < LOD |
| 9 | Urine* | < LOD | < LOD | < LOD | < LOD | < LOD | < LOD | < LOD | < LOD | < LOD | 1.1305 | < LOD | LOD<x< LOQ | < LOD |
| 10 | Urine* | < LOD | < LOD | < LOD | < LOD | < LOD | < LOD | LOD<x< LOQ | < LOD | < LOD | 0.6561 | < LOD | < LOD | < LOD |
| 11 | Urine* | < LOD | < LOD | < LOD | < LOD | < LOD | < LOD | LOD<x< LOQ | < LOD | < LOD | 0.3235 | < LOD | < LOD | < LOD |
| 12 | Urine* | < LOD | < LOD | < LOD | < LOD | < LOD | < LOD | < LOD | < LOD | < LOD | 0.6713 | < LOD | < LOD | < LOD |
| 13 | Urine* | < LOD | < LOD | LOD<x< LOQ | < LOD | < LOD | < LOD | < LOD | < LOD | < LOD | 0.3773 | < LOD | 0.2305 | < LOD |
| 14 | Urine* | < LOD | < LOD | < LOD | < LOD | < LOD | < LOD | < LOD | < LOD | < LOD | < LOD | < LOD | < LOD | < LOD |
