# Supplementary figures and images for: Multiple neonicotinoids in children’s cerebro-spinal fluid, plasma, and urine
Source: Environ Health. 2022 Jan 11;21:10. doi: 10.1186/s12940-021-00821-z (PMC8750865; doi:10.1186/s12940-021-00821-z)

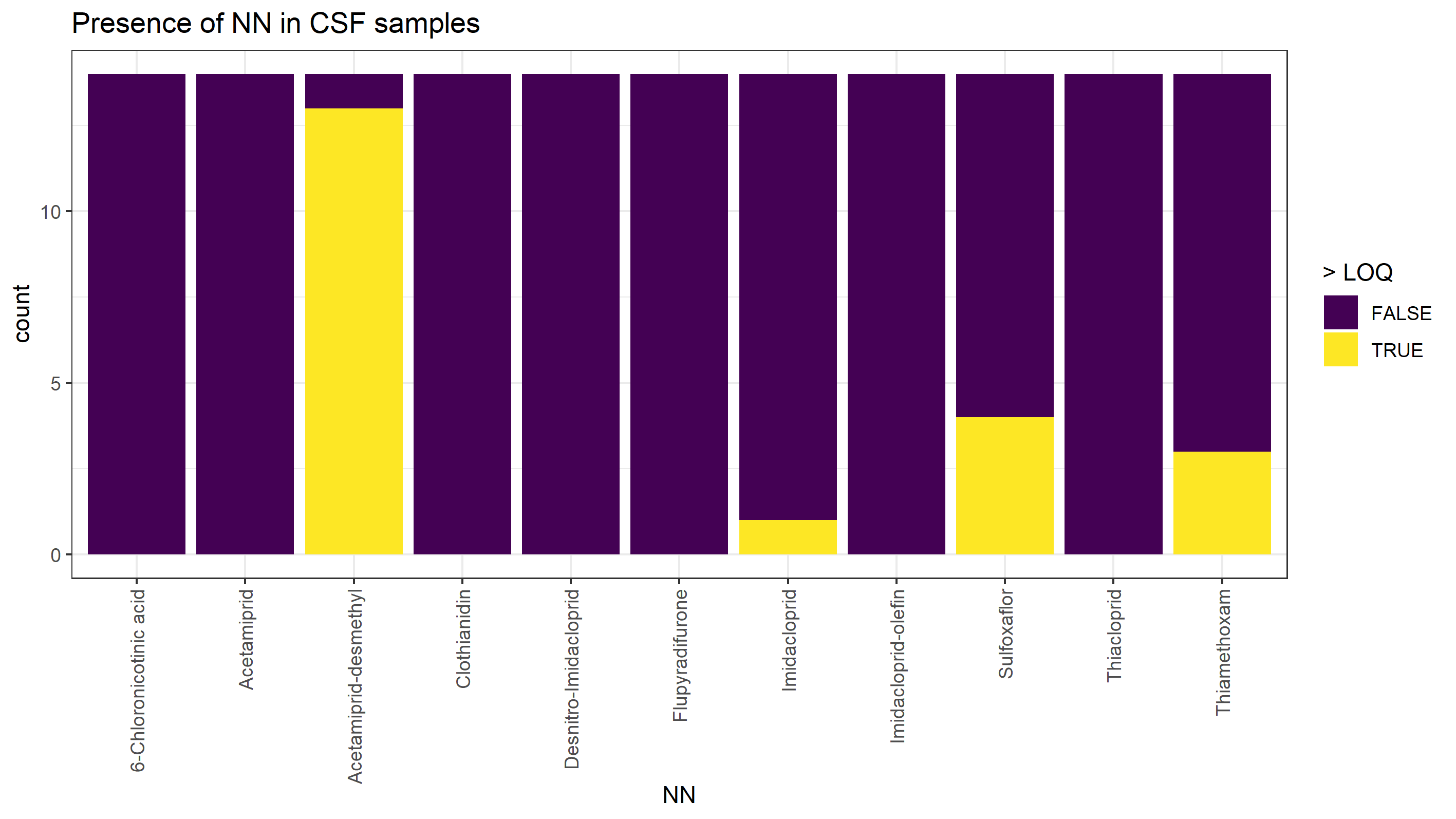

Supplement: Supplementary file 4 — Additional file 4: Supplementary Figure 1. (CSF occurence). Number of samples with neonicotinoids > lower limit of quantification, per neonicotinoid, in cerebrospinal fluid in 14 children with haematological cancers. NN, neonicotinoid; CSF, cerebro-spinal fluid; LOQ, lower limit of quantification. [file 12940_2021_821_MOESM4_ESM.png]

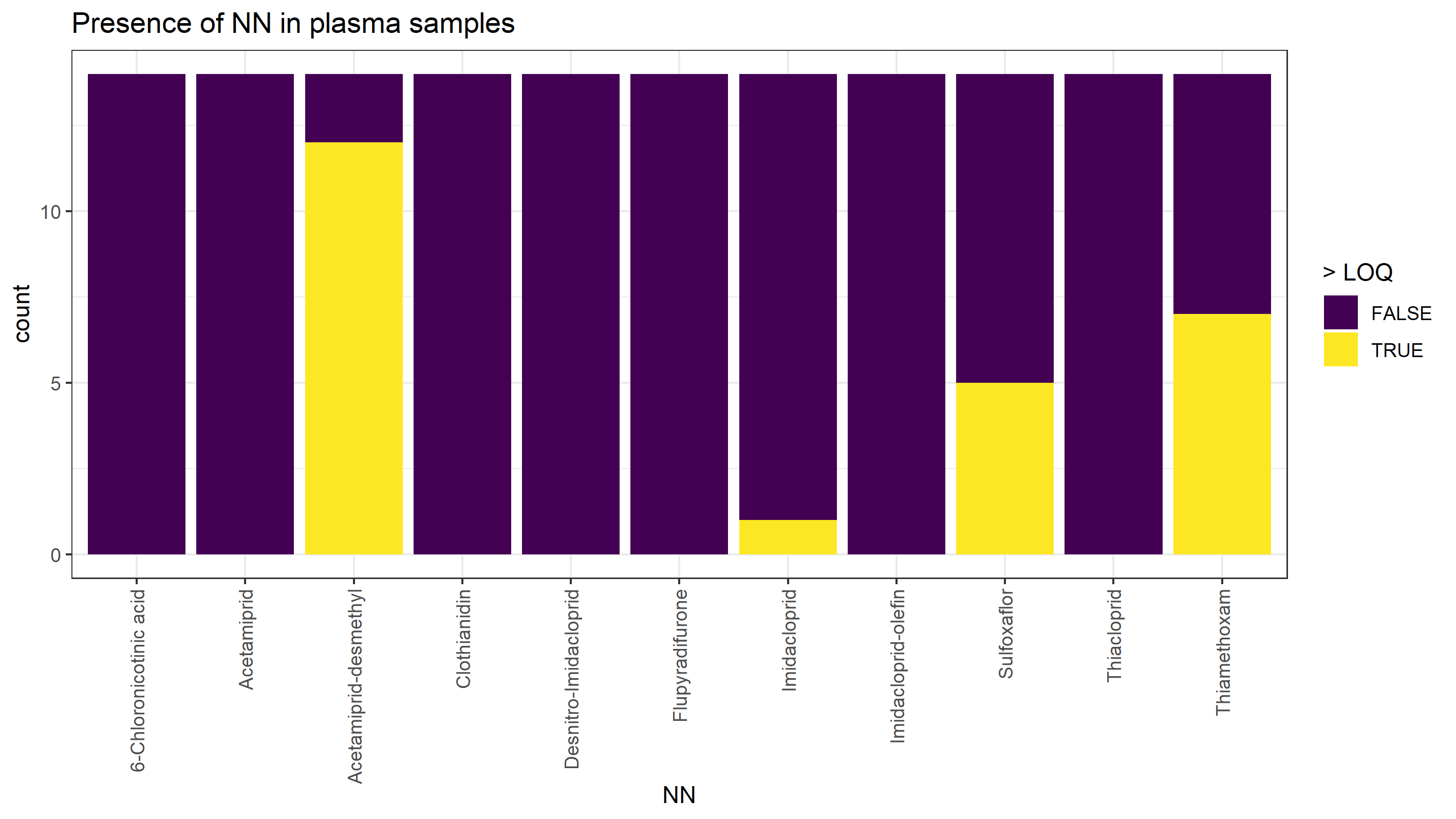

Supplement: Supplementary file 5 — Additional file 5: Supplementary Figure 2. (plasma occurence). Number of samples with neonicotinoids > lower limit of quantification, per neonicotinoid, in plasma in 14 children with haematological cancers. NN, neonicotinoid; LOQ, lower limit of quantification. [file 12940_2021_821_MOESM5_ESM.png]
